# Supplementary material for: A layered standards framework for integrating single-cell and spatial omics data into brain cell atlases
Source: bioRxiv. 2026 May 4:2026.04.30.722039. Preprint. [Version 1] doi: 10.64898/2026.04.30.722039 (PMC13174332; doi:10.64898/2026.04.30.722039)
Supplement: Supplement 3 [file media-3.pdf]

Step / Method	Tissue Sectioning	Sample prep / isolation	Optional Enrichment	Barcoding / tagging	Other Chemistry Steps	Imaging	Amplification	Adapter ligation & library completion	Methods References
**Single cell transcriptomic and multiomic assays**									
		TI → DC	DC → EC	EC → BC	na	na	BC → AC	AC → LI	
**Multiome (10x ATAC + GEX)**	na	Nuclei extraction from cells/tissues.	Optional FACS sorting to enrich cell populations	GEM encapsulation with barcoded beads capturing both mRNA and ATAC fragments (Droplet barcodes).	mRNA Capture — Reverse transcription in droplets with oligo-dT; ATAC — Tn5 transposition of open chromatin.	na	Pre-amplify cDNA & ATAC fragments; THEN split for library prep.	Illumina adapters + sample indices added during library PCR (for ATAC and GEX).	https://www.10xgenomics.com/blog/introducing-chromium-single-cell-multiome-atac-gene-expression
**snm3C-seq**	na	Nuclei extraction from cells/tissues.	Single nuclei sorted into plates with optional enrichment.	Cell barcode introduced (plate indexing).	Chromatin conformation capture + bisulfite conversion for methylome + proximity ligation for contacts.	na	PCR amplify bisulfite-converted contact/methylome fragments.	Adapters are part of PCR products ready for NGS.	Luo et al. (2019). *Nature Methods 1* 6 (10) 999-1006.
**snmCT-seq**	na	Cell / Nuclei extraction from cells/tissues.	Single nuclei sorted into plates with optional enrichment.	Cell barcode introduced (plate indexing).	RNA reverse transcription + bisulfite conversion for methylome without physical DNA/RNA separation.	na	PCR amplify barcoded mixed RNA/methylome molecules to build sequencing libraries.	Adapters included in amplified products, ready for sequencing.	Luo et al. (2018) *bioRxiv* 434845; doi: https://doi.org/10.1101/434845
**Droplet Paired-Tag**		Nuclei extraction from cells/tissues.	Optional FACS sorting to enrich nuclei	Combinatorial indexing of histone marks and transcripts with cell barcode (Droplet barcodes).	Capture of histone modification targets (antibody-Tn5 chemistry) + mRNA capture inside droplets.	na	Amplify both transcript and epigenetic (histone) fragments post-barcoding.	Sequencing adapters added during PCR/ligation steps.	Xie et al. (2023) *Nature Structural & Molecular Biology* 30, 1428-1433.
**Sequencing-based spatial transcriptomic assays**									
	TI → SE	SE → DC	DC	EC → BC			BC → AC	AC → LI	
**DBiT-Seq**	Tissue sectioned	Tissue mounted on slides	Incubation with antibody-derived DNA Tags for proteins of interest.	Two sets of orthogonal spatial barcodes delivered before or after RT via microfluidic channels, ligated in situ.	In situ reverse transcription of mRNA to cDNA, barcodes ligated to transcripts/targets.	Imaging of individual pixels for proteins of interest	Extract spatially barcoded cDNA, PCR amplify with sequencing primers.	After spatial barcoding, template switch adds handles, PCR completes adapters.	Liu et al. (2020) *Cell 1* 83 (6), 1665-1681.E18.
**Slide-tags_recon**	Tissue sectioned	Fresh-frozen tissue section mounted on bead array; nuclei remain in situ.	na	Spatial barcodes absorbed by nuclei in situ. Spatially tagged nuclei isolated; microfluidics capture and barcoding of individual nuclei.	na	na	Downstream single-cell library amplification (standard droplet scRNA or other).	Standard Illumina adapter addition during library prep.	Russel et al. (2024) *Nature* 625, 101-109.
**Imaging-based spatial transcriptomic assays**									
	TI → SE	SE → DC	DC	DC → BC					
**MERFISH**	Tissue sectioned	Fixed tissue mounted on slides	Optional nuclear staining, anatomic staining and imaging	Combinatorial optical RNA barcodes: sequential rounds of hybridization and imaging			na	na	Chen et al. (2015) *Science* 348, aaa6090. DOI:10.1126/science.aaa6090

Table S2. Mapping assay-specific methods to generalized experimental workflow steps. Assay-specific methods were mapped to a shared set of generalized experimental workflow steps (as shown in Fig. 2) to support standardized metadata representation across single-cell, multi-omic and spatial transcriptomics data types. Each row corresponds to an assay family, and each column represents a generalized process category in the metadata model.
